# Training load of newly recruited nurses in Grade-A Tertiary Hospitals in Shanghai, China: a qualitative study

**DOI:** 10.1186/s12912-022-01138-z

**Published:** 2023-01-11

**Authors:** Chan Yu, Jinxia Jiang, Minhui Zhong, Han Zhang, Xia Duan

**Affiliations:** 1 Department of Gynecology, Shanghai Key Laboratory of Maternal Fetal Medicine, Shanghai Institute of Maternal-Fetal Medicine and Gynecologic Oncology, Shanghai First Maternity and Infant Hospital, School of Medicine, Tongji University, Shanghai, China; 2 Emergency Department, School of Medicine, Shanghai Tenth People’s Hospital, Tongji University, 200072 Shanghai, China; 3 Department of Nursing, Shanghai Key Laboratory of Maternal Fetal Medicine, Shanghai Institute of Maternal-Fetal Medicine and Gynecologic Oncology, Shanghai First Maternity and Infant Hospital, School of Medicine, Tongji University, No. 536, Changle Road, Jing’an District, Shanghai, 200040 China; 4 Wuxi Children’s Hospital, Jiangnan University, Jiangsu, China

**Keywords:** Newly recruited nurse, Training load, Semi-structured interviews, Qualitative research

## Abstract

**Background:**

This study aimed to provide insight into the training load of newly recruited nurses in grade-A tertiary hospitals in Shanghai, China. The lack of nurses in hospitals across China has resulted in newly recruited nurses in grade-A tertiary hospitals in Shanghai having to integrate into the work environment and meet the needs of the job quickly; thus, they undergo several training programs. However, an increase in the number of training programs increases the training load of these nurses, impacting the effectiveness of training. The extent of the training load that newly recruited nurses have to bear in grade-A tertiary hospitals in China remains unknown.

**Methods:**

This qualitative study was conducted across three hospitals in Shanghai, including one general hospital and two specialized hospitals, in 2020. There were 15 newly recruited nurses who were invited to participate in semi-structured in-depth interviews with the purpose sampling method. A thematic analysis approach was used to analyze the data. The COREQ checklist was used to assess the overall study.

**Results:**

Three themes emerged: external cognitive overload, internal cognitive overload, and physical and mental overload.

**Conclusion:**

Through qualitative interviews, this study found that the training of newly recruited nurses in Shanghai’s grade-A tertiary hospitals is in a state of overload, which mainly includes external cognitive overload, internal cognitive overload, physical and mental overload, as reflected in the form of training overload, the time and frequency of training overload, the content capacity of training overload, the content difficulty of training overload, physiological load overload, and psychological load overload. The intensity and form of the training need to be reasonably adjusted. Newly recruited nurses need to not only improve their internal self-ability, but also learn to reduce internal and external load. Simultaneously, an external social support system needs to be established to alleviate their training burden and prevent burnout.

## Introduction

Newly recruited nurses are nurses who have begun clinical nursing work and are within two years after graduation [1]. They are the main human resources among the fresh recruits of a hospital, and the level of their nursing quality affects not only the quality of clinical nursing services but also patient safety [2]. However, upon beginning their clinical work, newly recruited nurses often realize that they lack the necessary experience, skills, and abilities to identify with and respond to patients. They are sometimes unable to perform various clinical nursing tasks, quickly adapt to changes in roles and environments, and meet clinical or medical needs.

The development of standardized training for such nurses has effectively compensated for this shortcoming and has played a key role in urging them to adapt to the new environment of clinical work, while simultaneously giving them sufficient time to develop their own abilities [3]. However, most studies focus on how to provide better training for new nurses, and less attention has been paid to the training load of the newly recruited nurses in China [4]. Moreover, some studies have found negative effects of training and indicated that some nurses sleep in training classes [5]. These reports have prompted nursing managers to think about the training load faced by new nurses, particularly whether too much training may affect nurses’ physical and mental health.

The Cognitive Load Theory states that based on the limited capacity of working memory and the constancy of total cognitive resources, the rational allocation of cognitive resources is the key to effective learning [4]. The core concept of Cognitive Load Theory points out that the form, content, time and frequency of training, any unreasonable design will cause cognitive overload of training objects, thereby affecting the training effect. In view of the need for systematic and standardized training at the beginning of the newly recruited nurses’ entry, the training load is also generated. Therefore, this study intends to use cognitive load theory as a guide to understand the current status of training load of the newly recruited nurses through qualitative research, analyze the influencing factors of training load, and provide reference for alleviating nurse training burnout and improving training quality.

## Background

Newly recruited nurses refer to nurses who have entered clinical nursing work within two years after graduation. They are the main human resource among hospital freshmen, and the level of their nursing quality affects not only the quality of clinical nursing services but also patient safety [6]. However, upon beginning their clinical work, newly recruited nurses realize that they often lack the necessary experience, skills, and abilities to identify with and respond to patients. They are unable to perform various clinical nursing tasks, quickly adapt to changes in roles and environments, and meet clinical or medical needs. The development of standardized training for such nurses has effectively compensated for this shortcoming and has played a key role in urging them to adapt to the new environment of clinical work, while simultaneously giving them sufficient time to develop their own abilities [3].

Standardized training of newly recruited nurses refers to the specialized training that nurses receive after completing their basic education from nursing colleges [7], which is an important transitional stage between school education and clinical work [8]. Simultaneously, as an essential component in the development of not only the nursing discipline but also the talent team, standardized training of newly recruited nurses is considered the “cradle project” of nursing talent training. This plays an important role in strengthening the construction of hospital nursing talents and improving the quality of nursing service [9]. At present, the standardized training of newly recruited nurses differs from one country to another. There are no uniform national and state training standards in the United States [10]. In the United Kingdom, newly registered nurses require training, but it is not uniform and varies from person to person [11]. In Australia, the new nurse training system is more advanced, comprising centralized training and standardized rotation training [11]. Japan has established a “Clinical Nursing Practice Competence Ladder Education System,” which focuses on training nurses on clinically necessary knowledge and technical and professional values in the first year of newly recruited nurses’ employment [11]. The Hong Kong District Nursing Institute was established by the Hong Kong Hospital Authority to work with hospitals to provide nurturing programs for newly graduated nurses [10]. Taiwan takes a phased, gradual growth approach to systematic training, wherein nurses participate in a “two-year nurse training program” and undergo “specialist nursing training” in order to become senior nursing staff with professional nursing ability [12].

In 2016, the National Health and Family Planning Commission organized and formulated the “Newly Recruited Nurses’ Training Program (Trial),” which clarified the scope of application, training objectives, training targets, training methods and time, training content and requirements, assessment methods and content, among other elements. In the National Nursing Development Plan (2016–2020), the National Health Planning Commission clearly highlighted “establishing a nurse training mechanism to enhance professional quality” and, in particular, establishing a “demand-oriented, job-based competence as the core” nurse training system [13]. In this context, medical institutions at all levels in China have further strengthened the training of newly registered nurses, which is mainly reflected in: (1) the training content being more abundant and diverse—from basic nursing to specialized disease nursing knowledge and skills to the present demands, covering laws and regulations, communication, etiquette, scientific research theories and methods, computer and informatization, price management, quality control, etc.; and (2) the training plan being more complete and standardized—from the previous lack of clear rotation requirements and specifications to the current requirements that at least four departments should be rotated, including internal medicine, surgery, intensive care, and emergency, each of which should be rotated for at least three months. However, the training effect is not satisfactory. When training is translated into actual work done by newly registered nurses, their performance and the organizational performance are low [14]. This inefficiency is usually manifested in actual training scenarios, such as nurses dozing off at work, playing with mobile phones, exhibiting no interest in training, and complaining about frequent training in clinical work. Simultaneously, the fact that the training content cannot be effectively transformed into clinical applications also indirectly reflects the same problems.

If this series of problems in the standardized training of newly registered nurses cannot be effectively solved, the goal of improving the professional quality and ability of nurses cannot be achieved, which will result in increasing management costs and wastage of resources. This has prompted managers to ponder the following questions: (1) How can we take into account the load bearing level of nurses’ learning, stimulate their interest in learning, and improve training effectiveness while strengthening training? (2) Can it be based on evaluations by others, from the perspective of nurses, to quantify the training load they receive at work, so that they can receive training that corresponds to their cognitive load, improve their learning enthusiasm, and promote their learning content to be better transformed into clinical applications ? Several studies have confirmed that continuous and intensive training can effectively improve the professional ability of nurses. However, whether the training increases nurses’ physical, psychological, and living burden is not clear [15].

Therefore, the purpose of this study is to shed light on the training load and its impact on newly recruited nurses in grade-A tertiary hospitals in Shanghai and identify some means by which they can cope with excessive training load to avoid job burnout.

## Methods

### Design

A qualitative study was conducted to investigate the training load of newly recruited nurses at grade-A tertiary hospitals in Shanghai, China. The method of qualitative research is consistent with the phenomenological method and is used to elicit the subjective feelings of the newly recruited nurses receiving standardized training in grade-A tertiary hospitals.

### Sample/participants

The participants were recruited from among the newly recruited nurses working in three hospitals of Shanghai, including one general hospital and two specialized hospitals which are affiliated with different universities. The eligibility criteria of newly recruited nurses for participation included: (1) being on-the-job registered nurses; (2) having worked less than 2 years; (3) being willing to participate in this study. The exclusion criteria included: (1) not being clinical nurses; (2) newly recruited nurses on leave during the survey period, and (3) trainee nurses. The researchers sent an invitation via email or WeChat to request a purposive sampling of newly recruited nurses to participate in individual interviews.

### Data collection

Semi-structured and face-to-face individual in-depth interviews were conducted between July and August 2020. The interview questions were based on the research conducted by McCalla-Graham and De Gagne [16]. The interviews began with initial questions about personal information, including regarding the current department in which the nurses were working and the previous work department in the hospital; they then moved on to explanatory questions, as shown in Table 1. The interviews were arranged by prior appointment and were conducted in a place where participants felt comfortable to freely express their experiences. The interviews took about 40 ~ 60 min due to the participants’ tolerance and willingness. The whole process of interviewing was recorded with participants’ knowledge and approval; the data were then transcribed, encoded, and analyzed immediately after each interview. Each interview continued until the topic was well formed and no new data was provided. When the interview with the 15th new nurse was completed, the data collection was terminated because no new information was uncovered.

**Table 1:** Semi-structured interview questions

No.	Questions
1	As a new nurse, what is the training you currently involved in the hospital?
2	What are your experiences and feelings about training provided by the hospital?
3	How do you evaluate the training in the hospital?
4	Has the training had an impact on your work and life? If so, what is the impact?
5	What are the main causes of the impact?

### Ethical consideration

Ethical approval was obtained from a grade-A tertiary hospital. All participants were informed about the aims and process of this study, signed written informed consent forms prior to being interviewed, and participated in this study voluntarily. The transcriptions were anonymized by using a number rather than the participants’ name to ensure their confidentiality. They were free to withdraw from the research at any point of time.

### Data analysis

Within 24 h of the interview, the data was transcribed verbatim from the recording and thematically analyzed using a six-step method. Thematic analysis is often used in studies to examine communication among various groups to identify the patterns within the data. In addition, because it is a data-driven approach, themes can be formed directly from the original data [17].

In order to analyze the obtained data, first, the researcher repeatedly listened to the interviews several times before they were transcribed. Second, the initial encoding was performed. Third, codes with similar meanings were listed under a sub-theme with an appropriate label. Fourth, the sub-themes with similar meanings were classified as a theme. Fifth, the initial codes, sub-themes, and themes were reviewed and relabeled. Finally, the report was written [18, 19].

To ensure the trustworthiness of the data, researchers were granted sufficient time to collect and go back and forth between the data to ensure that it was acceptable. In order to form the sub- and main themes, the initial codes were moved between the sub-themes several times. Dependability was achieved through the development of an audit trail and a qualitative expert reviewed the findings.

To enhance the credibility of the study, the transcripts and extracted codes were returned to the participants to ensure that the meanings matched their opinions. Peer debriefing was done with the qualitative expert not only to ensure that the findings emerged from the data, but also to gain new perspectives of the data.

### Rigor and quality criteria

To ensure the criteria of credibility, transferability and dependability [20–22] a series of actions was carried out: (1) a semi-structured interview protocol to ask the same questions to all participants was used; (2) the selection of participants ensured proximity to the phenomenon studied and a wealth of information; (3) the context and characteristics of the participants were reported on in detail; (4) the presentation of the findings was accompanied by abundant quotes from the participants’ discourse; (5) the interviews were recorded, transcribed verbatim and returned to obtain confirmation by participants to ensure the accuracy of the recorded data; (6) the analysis was carried out independently by two researchers and the entire research team participated in the consensus process, validating the results. A review system was established to allow the process to be replicated step by step. The execution and evaluation of the study were assessed with the COREQ qualitative design checklist.

## Results

We start with the first hospital and select one participant sequentially as the interviewee. And so on until the subject information is saturated. Data were collected from 15 participants (all female nurses) aged between 21 and 26 years, with work experience in three Shanghai hospitals, including one general hospital and two specialized hospitals. They comprised 1 postgraduate, 9 undergraduates, and 5 junior college students. All of them were single (Table 2).

The study findings extracted three themes (external cognitive overload, internal cognitive overload, physical and mental overload) and seven sub-themes, as described below (Table 3).

**Table 2:** Characteristics of participants (*n* = 15)

Number	Gender	Age (years)	Marital status	Hometown	Educational Background	Major
N1	Female	26	single	Guangxi	Master	Nursing
N2	Female	22	single	Shanghai	Undergraduate	Nursing
N3	Female	24	single	Shanghai	Undergraduate	Midwifery Nursing
N4	Female	22	single	Shanghai	Junior	Nursing and Health Management
N5	Female	24	single	Shandong	Undergraduate	Nursing
N6	Female	23	single	Shanghai	Undergraduate	Nursing
N7	Female	21	single	Shanghai	Junior	Nursing
N8	Female	22	single	Shanghai	Undergraduate	Nursing
N9	Female	22	single	Anhui	Undergraduate	Nursing
N10	Female	23	single	Anhui	Undergraduate	Midwifery Nursing
N11	Female	23	single	Guangxi	Undergraduate	Nursing
N12	Female	23	single	Jiangsu	Undergraduate	Midwifery Nursing
N13	Female	21	single	Zhejiang	Junior	Nursing
N14	Female	21	single	Guangdong	Junior	Midwifery Nursing
N15	Female	22	single	Gansu	Junior	Midwifery Nursing

**Table 3:** The Training Load of newly recruited nurses in China

Themes	Sub-themes
External cognitive overload	the form of training overload
	the time and frequency of training overload
Internal cognitive overload	the content capacity of training overload
	the content difficulty of training overload
Physical and mental overload	physiological load overload
	psychological load overload
	life load overload

### External cognitive overload

The external cognitive overload theme results from improper teaching design and teaching activities; it comprised two sub-themes: the form of training overload, and the time and frequency of training overload.

#### The form of training overload

Most newly recruited nurses revealed that the current training method was monotonous and time-consuming, with concentrated training hours. One participant expressed, *“Centralized traditional classroom training in the hospital is the usual form; PowerPoint (PPT) is used as the main means to teach us and the teachers always read out the PPT to us. This makes it difficult to concentrate and easy to get distracted.“* (N4). Another said, “*The training has become a formality; nobody guides us and nobody gives us the tracking of and feedback from the training effects.”* (N3).

#### The time and frequency of training overload

The majority of newly recruited nurses believed that the training load was related to the management staff’s failure to allocate time for rest and arrange tasks. One participant pointed out, *“Sometimes I will go out for training during night shifts, and I’m so confused—always distracted during training.“* (N9).

The training frequency is unreasonable, resulting in increased training pressure, making it difficult to achieve the expected value. One participant stated, *“There is a lot of training now. On average, there is theoretical training and practical training every month. In addition, sometimes there are related legal training, nursing ward round training, and emergency drill training. I feel like I have been participating in the training; I’m under great pressure.“* (N1).

### Internal cognitive overload

The internal cognitive overload theme describes the characteristics of the learning content itself and the knowledge level of the learner; it comprised two sub-themes: the content capacity of training overload and the content difficulty of training overload.

#### The content capacity of training overload

The training content is very rich, making it difficult to remember and absorb in a short time. If the cognitive resources cannot be allocated reasonably, when the total cognitive resources required in the training exceed the total working memory resources, it will cause cognitive overload for newly registered nurses. One participant stated, *“Sometimes it is necessary to undergo both theoretical and practical training in one day. After training, I have to take an exam. I feel that my mental resources are inadequate.“* (N3).

On the other hand, a higher educational background requires further training and, because the training content is more diversified, the training load will also increase correspondingly. One participant stated, “*My roommate undergoes more training than I do. She is a master’s graduate, and sometimes the department leader asks her to participate in some training on scientific research*.” (N15).

#### The content difficulty of training overload

The lower the educational background, the heavier the training load of clinical nurses. Faced with the same training content, nurses with lower academic qualifications find it more difficult to learn, thereby feeling the load of the higher level of training over time. Some nurses in this study have a college degree, and their knowledge level is no match for the difficulty and scope of the training content, causing them to feel a higher content load during training. One participant pointed out, *“I am a junior college student. We used to focus more on operations in school. Theoretical knowledge is relatively weak—not as good as that of the undergraduates. Sometimes the teacher talks about pathogenesis, etc., but I don’t understand.“* (N7).

Newly registered nurses with more clinical practice experience felt a lower training load, needed less time to deal with basic clinical nursing work, and had enough energy to study independently. One participant stated, *“I think the current training is still acceptable. When I was in college, I had a very fulfilling life every day. I not only had to complete my usual studies, but also actively participate in social practice activities, such as going to the community to measure blood pressure for the elderly and going to a rehabilitation hospital to assist patients with rehabilitation exercises.“* (N11).

### Physical and mental overload

The physical and mental overload theme consisted of three sub-themes including physiological overload, psychological overload, and life overload.

#### Physiological overload

Physiological load overload is mainly manifested in decreased enthusiasm for work and fatigue among newly recruited nurses, which affects the quality of work and even leads to resignation. One participant stated, *“Sometimes when I think of training tomorrow and having to take an exam after the training, I can’t sleep; one time, I was in training in my dream at night. Therefore, the next day at work, the whole person is a little in a trance.”* (N8). Another participant expressed, *“I’m so tired; no matter how long I think about going to the hospital for training tomorrow, I don’t want to go to work, and sometimes I even think it’s better to change to a job without training.”* (N12).

#### Psychological overload

Psychological load overload is mainly manifested in newly recruited nurses often feeling nervous and anxious, which affects their sleep and work status. One participant pointed out, “*Sometimes when I think of going to the hospital for training during night shifts or on breaks, I feel very anxious.“* (N14). Another participant stated, *“I feel nervous at the thought of the upcoming exam. I keep thinking about it, even when I go to work and when I sleep. It seriously affects my sleep, which dampens my spirit and mood at work the next day*.*”* (N9).

#### Life overload

Life load overload is mainly manifested in the fact that newly recruited nurses have no time to fall in love and cannot spend time with their families, thus losing interest in work. One participant expressed, *“I feel that ever since I went to work, I have no time to spend time with my family, no time to fall in love, and I don’t even know why I go to work.”* (N2).

## Discussion

This study describes the training load of newly recruited nurses in grade-A tertiary hospitals in China. Three extracted themes can be applied to research on nursing training for newly recruited nurses. The findings from this study reveal that there is an external cognitive overload for newly recruited nurses in grade-A tertiary hospitals in China, as reflected in the concentrated class hours and monotonous and time-consuming training methods. Moreover, training is often a mere formality, with training management lacking orientation and traction, and ignoring the tracking and feedback of the training effects. These findings are consistent with Wang’s research results [23]. The singular presentation of materials and resource forms will inevitably generate additional form load [24]; thus, we should effectively reduce the training load through the informatization and diversity of training forms. Research scholars have proposed “just-in-time training” based on mobile technology, which can effectively reduce the training load [25]. The application of new training modes based on the network platform can enhance the effectiveness of clinical training [26, 27]. Simultaneously, the generation of training load is related to the management staff’s failure to arrange tasks effectively and allocate time for nurses to rest [4]. The training time and frequency are unreasonable, resulting in increased training pressure and difficulty achieving the desired training effect. In this study, this is reflected in nurses playing with mobile phones during training, distracting themselves, complaining about frequent training, the overlap between training time and rest time, and the duplication of learning content in clinical work. It also reflected indirectly in the training content not being effectively transformed into clinical applications, which is a similar finding to Dong’s research [14]. Nursing managers need to arrange the time and frequency of training reasonably based on the actual hospital situation.

This study reveals that the internal cognitive capacity of newly recruited nurses in grade-A tertiary hospitals in China is overloaded. Some nurses who participated in the survey have a college degree, and their knowledge level is inadequate to cope with the difficulty and scope of the training content, which causes them to feel a higher content load during the training process. Similar findings have been observed in other studies [28]. Cognitive load theory proposes that internal cognitive load depends on the complexity of learning content and the learner’s original knowledge level [25]. At present, the main problem is that although the training content is rich, if the cognitive resources cannot be allocated reasonably, the total amount of cognitive resources required in the training will exceed the total amount of working memory resources, which will lead to cognitive overload [29]. Hospital managers can decompose training objectives and dynamically display training content, taking full account of the limitations of individual working memory capacity [30]. Moreover, they can clearly define key training content, effectively avoiding repeated training on core systems, nursing routines, and other basic theories.

The study also shows that the physical and mental capacity of newly recruited nurses in grade-A tertiary hospitals in China is overloaded. Establishing an external social support system can help relieve the physical and mental burden of newly recruited nurses. Social support from family is the main external impetus for newly recruited nurses to improve their career adaptability, as they transition from campus to society, requiring support from family, friends, and colleagues in all aspects of life and work. The care, guidance, and encouragement of colleagues and seniors working in the hospital are also powerful reserves for newly recruited nurses’ career adaptability.

### Study limitations

Although the study interviewed 15 participants and achieved data saturation, the interviews were conducted in only three tertiary hospitals in Shanghai, and the source of sample data was extremely limited, so the findings from this study may thus not be transferable other contexts. In the future, newly recruited nurses can be added to include data from different hospitals across the country to further validate the results of this research. However, this study provides information with regard to the training load of newly recruited nurses.

## Conclusion

Nursing is a comprehensive subject. Newly recruited nurses need to grasp a lot of knowledge in medicine, nursing, humanities and sociology, and informatics. Continuous or intensive training, allows newly recruited nurses to acquire a lot of knowledge as soon as possible. Although many studies have confirmed this, there is little research examining whether large and frequent training causes training load for newly recruited nurses. Through qualitative interviews, this study found that the training of newly recruited nurses in Shanghai’s grade-A tertiary hospitals is in a state of overload, which mainly includes external cognitive overload, internal cognitive overload, physical and mental overload, as reflected in the form of training overload, the time and frequency of training overload, the content capacity of training overload, the content difficulty of training overload, physiological load overload, and psychological load overload. Therefore, it is recommended that nursing managers learn cognitive load theory, set training content scientifically, arrange training time and frequency reasonably, and learn from new domestic and foreign training methods, such as standardized patient training models, micro-classes, and massive online open courses. The training of newly recruited nurses is carried out in different ways to mobilize their initiative to participate in the training, to improve the effectiveness of training.

## Data Availability

All data generated or analysed during this study are included in this published article.
